# Comparison between Smokers and Smokeless Tobacco Users in Their Past Attempts and Intentions to Quit: Analysis of Two Rounds of a National Survey

**DOI:** 10.3390/ijerph192013662

**Published:** 2022-10-21

**Authors:** M. Mofizul Islam

**Affiliations:** Department of Public Health, Room 410, Health Sciences Building 2, La Trobe University, Bundoora, VIC 3086, Australia; mofi.islam@latrobe.edu.au; Tel.: +61-03-9479-2639; Fax: +61-03-9479-1783

**Keywords:** smoking, smokeless tobacco, global adult tobacco survey, quit attempts, cessation, Bangladesh

## Abstract

This study compares current tobacco smokers and smokeless tobacco (SLT) users in terms of their past quitting attempts and intentions to quit in the future, and identifies approaches used in their recent quitting attempts. Data (*n* = 14,498) of current tobacco users from two rounds of the Global Adult Tobacco Survey Bangladesh were analysed. Poisson regressions with robust variance were used to examine associations between the study factor and the two outcome variables. About half of smokers and a quarter of SLT users tried to quit during the 12 months before the survey. About two-thirds of smokers and half of SLT users intended to quit in the future. Smokers were more likely (adjusted prevalence ratio (aPR): 1.38, 95%CI: 1.24–1.53) than SLT users to have attempted to quit during the 12 months before the survey and to intend to quit in the future (aPR: 1.09, 95%CI: 1.02–1.16). The corresponding aPRs were even higher for dual users (smoked tobacco and used SLT). Future intention to quit for both smokers (aPR: 1.45, 95%CI: 1.38–1.53) and SLT users (aPR: 1.87, 95%CI: 1.76–1.98) was significantly associated with their past quitting attempts. Most of those who had attempted to quit did not receive any treatment. Proactive and tailored interventions to promote quitting and expansion of tobacco cessation methods are recommended.

## 1. Introduction

Tobacco smoking and smokeless tobacco (SLT) use are widespread in most South and Southeast Asian countries, including Bangladesh. Bangladesh is the 11th largest tobacco-producing country [1]. The prevalence of smoking and SLT users are relatively high in Bangladesh [2,3], and so are the adverse health outcomes. It was estimated that in 2016 alone, tobacco use caused more than 160,000 deaths, which could have been prevented with appropriate interventions [4]. Thus, to drastically reduce tobacco use, the government set a target to turn Bangladesh into a tobacco-free country by 2040. In recent years, Bangladesh initiated several large-scale programs to substantially reduce tobacco use. It is one of the first signatory countries of the WHO Framework Convention on Tobacco Control (FCTC). The tobacco control measures received much-needed momentum after signing this convention, which is evident from recent surveys on tobacco use. For instance, the Global Adult Tobacco Survey (GATS) Bangladesh suggests smoking prevalence declined from 23% in 2009 to 18% in 2017, and SLT prevalence declined from 27.2% in 2009 to 20.6% in 2017 [4]. These declines can be attributed, in part, to the increasing tobacco cessation by the users and highlight the importance of promoting cessation services. Indeed, implementing tobacco cessation programs along with tobacco control measures has a synergistic effect and can maximise their impact at the population level [5]. To deliver the required cessation services, it is critical to apprehend the differences between the smokers and smokeless tobacco users in terms of their past initiatives and future intentions to quit, and the diverse underlying factors associated with that. It is also crucial to understand the methods for cessation these two user groups pursue.

Cessation of tobacco use is a complex process and often requires multiple attempts, with a high relapse occurring [6,7]. Past attempts to quit smoking or intentions to quit in the future are known to be significantly associated with successful cessation [8,9,10]. Moreover, quitting intentions and their stages and subsequent quit attempts are substantial predictors of ultimate success in cessation, and they vary across the types of tobacco use [11]. There could be several reasons for this variation, including the perceived or real differences in adverse health effects of different types of tobacco use, social acceptability, and inadequate treatment support for certain tobacco types. For instance, in many countries, compared to tobacco smoking, SLT use is considered less harmful to health [12]; also, unlike tobacco smoking, SLT use does not have second-hand exposure. Inadequate treatment support for SLT cessation may also, to some extent, influence the frequency of intention to quit attempts [12]. However, almost all published data on quitting attempts are about tobacco smoking. Little is known about the past quitting attempts or future intention to future quit attempts among smokeless tobacco users, and how this compares to smokers. The comparative data that exist are mostly from developed countries or are seldom the focus of the papers [13]. Findings from studies conducted in some Southeast Asian countries suggest that the determinants of quitting are only partly consistent with those found in Western countries [14].

Moreover, the intention to quit is known to be significantly associated with the previous quitting attempts and their duration [8,15,16]. There are several explanations for the relationship between past attempts and future intention to quit. For instance, tobacco users with a history of quitting attempts may be in the preparation stage and others without a quitting history are in the contemplation stage [17]. Past quitting experience also regulates an individual’s attitudes and beliefs about another future quitting intention [18]. Additionally, people who attempted to quit are likely to have higher self-efficacy than those without a quit history. The momentum that initiated the previous quitting attempt may be temporally persistent and thus continues to influence further quitting intentions [19]. Again, this relationship between past quitting attempts and future intention to quit may vary with the types of tobacco an individual use, while other things remain the same. However, little is known about this, particularly regarding smokeless tobacco use.

Furthermore, evidence suggests that the availability and accessibility of tobacco cessation services and related support play critical roles in overall quitting attempts and thus ultimate success [20]. Although some treatment services may be available for smoking cessation in most low- and middle-income countries, treatments for SLT-use cessation are scarce. Accordingly, while some previous studies examined treatment supports for those who attempted to quit smoking, data are scarce about the approaches SLT users employ in their cessation endeavours. This study aims to examine the past attempts and future intention to quit tobacco smoking, estimate their associations with the types of tobacco users (smokers, SLT users, and dual users), and describe the approaches that the smokers and SLT users used during their recent quitting attempts.

## 2. Materials and Methods

### 2.1. Study Participants

We used data from the recent two rounds of the Global Adult Tobacco Survey (GATS) in Bangladesh. GATS is a household survey of nationally representative people aged 15 years or older. It uses country-specific, stratified, multi-stage cluster sampling. A two-stage stratified sampling process was adopted in Bangladesh. At the first stage, administrative divisions were selected, followed by further stratification of rural and urban enumeration areas (EAs) within each division. The Bangladesh Bureau of Statistics developed those EAs for the national census. A total of 400 EAs were selected in 2009 and 496 EAs in 2017, with equal allocations of EAs to each administrative division and urban and rural stratum [4,21]. In the second stage, households’ listing and mapping operation was carried out in all the selected EAs and this list was used as the sampling frame. The tractional interval technique was used to select 30 households systematically from each EA, with an equal probability. Finally, one individual was randomly selected from all eligible people living in the participating households. The survey methods in both rounds were largely identical, which helped to compare the results. The total number of participants was 9629 in 2009 and 12,783 in 2017. The overall response rates were 93.6% and 93.8%, respectively. The questionnaire covered a range of items, including the demographic characteristics of the participants, households’ wealth status, the history of tobacco use, frequency and types of smoke and smokeless tobacco use, previous attempts and future intentions to quit, perception of the health effects of smoking and SLT use and second-hand smoke, and observations about the media coverage of tobacco prevention measures. Handheld electronic devices were used to record responses. The field supervisors conducted spot checks to ensure the validity and accuracy of the data collection. Bengali versions of the questionnaire were programmed and used on handheld devices. More details of the data collection can be found in the GATS reports [4,21].

### 2.2. Dependent and Independent Variables

This study has four dependent variables: (i) quitting attempts regarding smoking or SLT use in the 12 months prior to the interviews; (ii) intention to quit smoking or SLT in the future; (iii) intention to quit smoking in the future; and (iv) intention to quit SLT in the future. Each of these four outcome variables has two response categories, “yes” and “no”. Quitting attempts were examined by asking the participants if they made any attempts to stop smoking or SLT use during the past 12 months prior to the survey. Participants whose quitting attempts lasted for less than one day were also counted. However, the duration of the quitting attempts was months, weeks, and days for 88% of the smokers and 81% of the SLT users who attempted to quit in the 12 months prior to the survey.

The primary independent variables (study factors) are as follows: (i) type of smoking, with three categories, namely, SLT, smoking and both SLT, and smoking (dual use); (ii) attempt to quit smoking 12 months before the surveys; and (iii) attempt to quit SLT in the 12 months before the surveys. A range of other independent variables was used in the analysis. They are user type (occasional user, daily user), participants’ age, sex, educational status, professions, wealth quintiles of the households, places of residence (urban and rural) and administrative division of their usual residence, perceptions of whether tobacco is harmful to health and received counselling from healthcare providers, nicotine replacement therapy, and Quitline and/or traditional treatment (yes, no). These variables were identified as potential confounders in the literature. Household wealth status was determined using principal component analysis of the asset information of several items that a household owned/availed, including bikes, fixed telephones, cellular telephones, televisions, sewing machines, refrigerators, washing machines, flush toilets, electricity connections, as well as the type of main material used for the roof of the main house (cement, tin, or bamboo/thatched/straw).

### 2.3. Other Variables

The other variables examined were quitting duration and approaches used by the smokers and SLT users who had tried to quit during the 12 months prior to the survey. The response options included attempting to quit without assistance, counselling from healthcare providers, switching to SLT (a response offered only to current or past tobacco smokers), receiving nicotine replacement therapy (NRT), and using Quitline or a telephone support line.

### 2.4. Data Analysis

Descriptive analysis was conducted to estimate the prevalence and types of tobacco users, their distributions across the demographic characteristics and other related variables, and the duration of abstinence among the participants who attempted to quit smoking or SLT use during the 12 months before the survey. Multilevel Poisson regression with robust variance was used to estimate the associations between the primary study factor (i.e., types of tobacco use) and the four outcome variables. We used Poisson regression (with robust variance) because the odds ratio from logistic regression from a cross-sectional study may significantly overestimate the relative risk when the outcome is common (e.g., prevalence > 10%) [22,23]. The prevalence of all four outcome variables were more than 10%. Four regression models were developed to examine the association between the study factors and four outcome variables. They were to examine the associations between

(i) quitting attempts regarding smoking or SLT use in the 12 months prior to the surveys (outcome variable) and the type of tobacco use (study factor);
(ii) intention to quit smoking or SLT use in the future (outcome variable) and the type of tobacco use (study factor);
(iii) intention to quit smoking in the future (outcome variable) and attempts to quit smoking in the 12 months before the surveys; and
(iv) intention to quit SLT use in the future (outcome variable) and attempts to quit SLT use in the 12 months before the surveys.

All regression models were adjusted for the potential confounders mentioned in the earlier section. Regression results were estimated as the adjusted prevalence ratio (aPR) and 95% confidence intervals (95% CI). Two-sided tests were used for all analyses and a *p*-value < 0.05 was considered statistically significant. Sample weights were applied in all analyses. We used Stata (version 15.2) for all analyses and Microsoft Excel for developing graphs.

## 3. Results

The prevalence of current smokers was 23% in 2009 and 18% in 2017 (Table 1), and the corresponding prevalence for SLT users was 27.2% and 20.6% in the two rounds, respectively. The average age at which smokers had initiated smoking was slightly more than 18 years and remained almost the same in both rounds of the survey. However, the average age of initiation of SLT use had increased from 26.6 years in 2009 to 28.4 years. Almost all smokers were men; only a small proportion was women (3.5% in 2009 and 2.2% in 2017). Slightly more than half of the SLT users in 2009 were women (14.1/27.2 = 52%) compared to 62% in 2017. People who received no formal education were the dominant users of both forms of tobacco use. The vast majority of tobacco users lived in rural areas; in 2009, around 75% of smokers lived in rural areas, compared to 78% in 2017. This figure for SLT users in rural areas was 75% in 2009 and 82% in 2017. The proportions of smokers and SLT users across the variables are presented in Table 1.

Around 45% of smokers in 2009 tried to stop smoking in the past 12 months, compared to 43% in 2017. Among SLT users, this proportion was almost 27% in 2009 and 30% in 2017. In 2017, this proportion was slightly reduced to around 43% among smokers while increasing to 30% among SLT users (Table 1). In the combined sample, 15.5% of current dual users attempted to quit both smoking and SLT use, 34.6% to quit only one form (i.e., either smoking or SLT), and the remaining 49.9% to neither form of tobacco in the 12 months prior to the survey. In the aggregate sample, 30.7% and 3.9% of the current dual users attempted to quit smoking only and SLT use only, respectively, in the 12 months prior to the survey (results not shown in the table). Sixty-eight percent of smokers in 2009 intended to quit smoking in the future, compared to 66% in 2017. Among the SLT users, the future intention to quit was around 49% and 51% in the two rounds, respectively.

**Figure 1:**
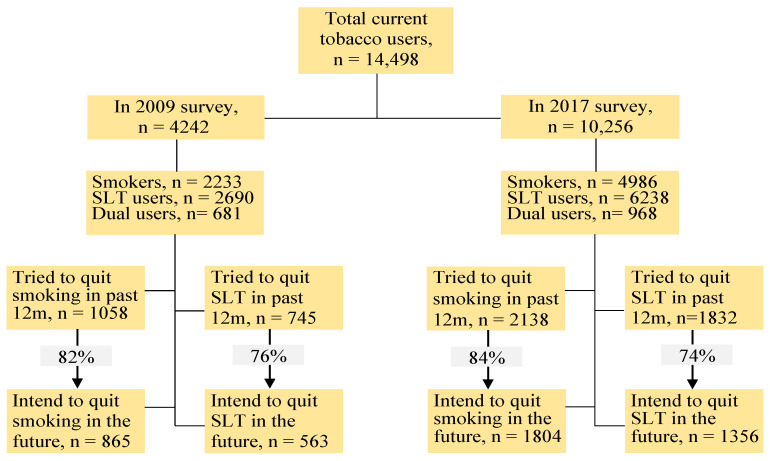
Flowchart of the two rounds of survey participants, presenting the total and two types of tobacco users, their quitting attempts in the 12 months before the survey, and their intentions to quit in the future. Note: SLT—smokeless tobacco; 12 m—12 months.

In the combined sample, 50.6% of current dual users intended to quit both smoking and SLT use, 21.3% to only one form (i.e., either smoking or SLT), and the remaining 28.3% to quit neither form of tobacco use in the future. In the aggregate sample, 16.2% and 5.1% of the current dual users intended to quit only smoking and only SLT use, respectively, in the 12 months prior to the survey (results not shown in the table). More than 80% of current smokers who had attempted to quit during the 12 months before interviews expressed an intention to quit in the future (Figure 1). Among the current users of SLT, approximately 75% of those who had attempted to quit during the 12 months before the survey expressed an intention to quit in the future. The prevalence of future quitting intention was approximately 54% in 2009 and 55% in 2017 among the current smokers who did not attempt to quit in the 12-month before the survey (results not shown in table). The corresponding figures for intention to quit SLT were 37% and 45% in the two rounds, respectively.

Table 2 presents the adjusted prevalence ratios of quitting attempts in the past 12 months and intention to quit in the future for smokers and dual users compared to SLT users, and the statistical associations of key factors with the two outcomes. Smokers were 1.44 and 1.10 times more likely than SLT users to have attempted to quit in the past 12 months and intend to quit in the future, respectively. The corresponding adjusted prevalence ratios for past quitting attempts and future intention to quit were 1.65 and 1.20 for the dual users, respectively. Daily users of tobacco were significantly less likely to report a quitting attempt in the 12 months before the survey (aPR = 0.79, 95%CI: 0.67–0.93) and intention to quit in the future (aPR = 0.76, 95%CI: 0.71–0.82). Users of the age group 65 years or higher were less likely than the 15–24-year age group to intend to quit in the future (aPR = 0.82, 95%CI: 0.74–0.92). Female users were less likely than male users to intend to quit in the future (aPR = 0.62, 95%CI: 0.57–0.67). Those who had completed secondary and further education or belonged to the high or highest wealth quintiles were more likely to intend to quit in the future. Participants who believed that tobacco use was harmful to their health were more likely than their counterparts to have attempted to quit in the past 12 months (aPR = 1.21, 95%CI: 0.94–1.56) and report that they intend to quit in the future (aPR = 1.21, 95%CI: 1.01–1.42), although the association was significant for the latter outcome only. Rural participants were less likely (aPR = 0.94, 95%CI: 0.87–1.00) than their urban counterparts to have attempted to quit in the past 12 months. The higher the age of initiation of daily use of tobacco by the participants, the more likely they were to report future intentions to quit.

**Figure 2:**
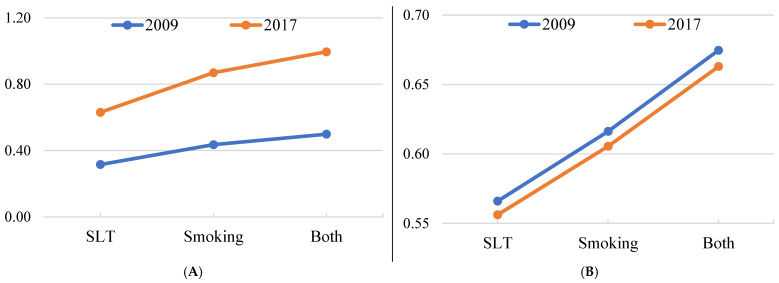
Predictive probabilities of (**A**) quitting attempt in the 12 months prior to the survey and (**B**) intention to quit in the future after two rounds of the survey.

Figure 2 presents the predictive probabilities of the quitting attempts in the 12 months prior to the survey and intention to quit in the future after two rounds of the survey. For both outcomes, the probabilities are higher in 2017 than in 2009, although the changes between the years were much higher for attempts made in the 12 months prior to the survey than for the intention to quit in the future.

Table 3 presents the adjusted prevalence ratios of intention to quit smoking or SLT in the future and quitting attempts during the 12 months before the survey, and the statistical associations of key factors with these two outcomes. Smokers and SLT users who attempted quitting in the 12 months prior to the survey were significantly more likely to report having an intention to quit in the future. Again, daily users were significantly less likely to report an intention to quit smoking (aPR = 0.81, 95%CI: 0.75–0.88) or SLT use (aPR = 0.80, 95%CI: 0.68–0.95) in the future. The relationship between these two outcomes (i.e., intention to quit smoking and SLT use in the future) with the socio-demographic factors remain mostly similar to the models presented in Table 2, except that the smokers living in rural areas were more likely (aPR = 1.07, 95%CI: 1.01–1.14) to express their intention to quit in the future.

**Figure 3:**
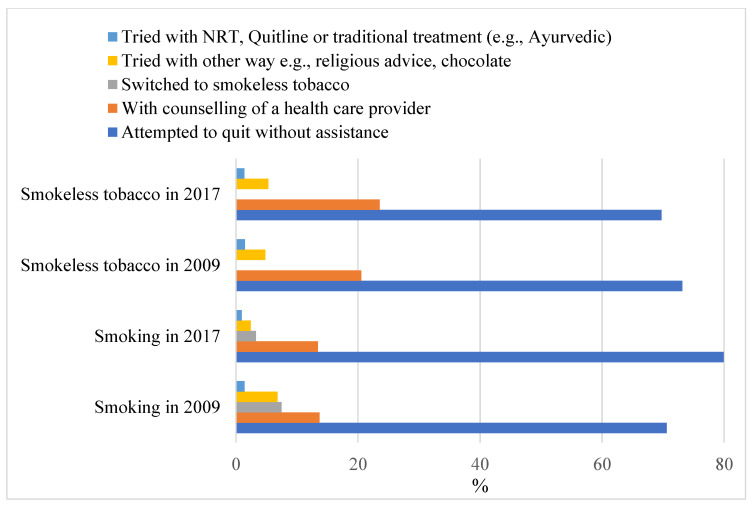
Various methods used by the tobacco users who attempted to quit in the past 12 months.

On average, exclusive smokers who attempted to quit in the 12 months prior to the survey abstained almost 35 days before experiencing recurrence. This figure for exclusive SLT users was almost 32 days. The corresponding figures for abstaining from smoking and SLT use were similar for the dual users. Among those who had endeavoured to quit in the 12 months prior to the survey, attempting to quit without the help of any treatment method was the dominant approach for both forms of tobacco users, followed by counselling from healthcare providers (Figure 3). Around 7.5% of smokers in 2009 and 3.3% in 2017 switched to SLT as a quitting approach to smoking. Only a small number of users had accessed NRT or used Quitline.

## 4. Discussion

This study provides a detailed picture of the quitting attempts among current tobacco users and identified the associated factors of past attempts and future intention to quit. The results of this study suggest that smokers were significantly more likely than SLT users to make an effort to quit in the past 12 months and intend to quit in the future. The prevalence is even higher for dual users than for smokers or SLT users. The results also suggest the prevalence of smokers who attempted to quit in the past 12 months decreased slightly between the survey rounds and increased to a similar extent among the SLT users (Table 1). The trends in intention to quit in the future among both smokers and SLT users remain similar to past attempts, although the overall prevalence is much higher. The results also suggest that tobacco users who had made quitting attempts in the 12 months before the survey were more likely to hold an intention to quit in the future. The vast majority of smokers and SLT users who had attempted to quit did not receive any treatment. Relatively more SLT users than smokers received counselling from healthcare providers in their attempts to quit and a small percentage received pharmacological or other treatments.

Our results suggest that almost half of the smokers and almost a third of SLT users made attempts to quit in the 12 months before the survey. Similarly, two-thirds of smokers and more than half of SLT users intend to quit in the future. Perhaps, the most crucial observation is that having an intention to quit in the future is significantly higher among those who attempted to quit in the 12 months before the survey. These observations are of substantial importance for the country’s tobacco cessation endeavour and warrant increased services and support for promoting quitting attempts while making varieties of cessation treatments and assistance available. At a population level, the rate of successful cessation of tobacco use is driven by two factors: The prevalence of quitting attempts and the prevalence of successful quitting among those who make a quitting attempt [24]. Thus, increasing the quitting attempts is an essential intervention to raise the “population quit rate”. Most tobacco users make multiple quitting attempts before succeeding; it was estimated that smokers make as many as 30 attempts on average [7]. Thus, it is critical to continuously motivate tobacco users to think about quitting and to make repeated quitting attempts, even if an attempt fails to succeed [25].

Although Bangladesh has made remarkable progress in raising awareness, there is a prevailing misconception among a subset of citizens that smokeless tobacco use has little or no adverse health effects [4]. Indeed, some people in rural areas believe that SLT use can help reduce pain, cure tooth and stomach aches, and the digestion process if taken after a meal, as well as help coping with boredom and frustration. The list of myths and misconception is long and varies across rural areas [26,27]. Perhaps this explains the significantly higher likelihood of past attempts and future intention to quit among smokers than SLT users. This observation is consistent with a previous study [13], in that those who believe tobacco use is harmful to health are more likely than others to report that they intend to quit in the future. A recent study found that relevant legislation remains ineffective in relation to graphical health warnings on SLT packets [28]. Appropriate measures need to be taken without further delay, along with specific programmes targeting SLT use to make people aware of its harmful effects.

We found women were significantly less likely than men to have intentions to quit tobacco use in the future in both rounds of the survey. This observation is consistent with the result of a study conducted in India [29], and is likely attributed to women’s low prevalence of tobacco smoking and high prevalence of SLT use. In Bangladesh, it is not socially acceptable for women to smoke. However, SLT use does not have any such prejudice, and it is considered normal for women. As a result, women do not feel social pressure to quit their SLT use. There may be other factors for women’s relatively low intention to quit SLT use, such as inadequate awareness of its adverse effects. Further research is needed to understand those factors and the barriers they face in quitting.

Our results suggest that the vast majority who had attempted to quit did not receive any treatment, and a small proportion had switched to SLT. One reason why so many tobacco users did not receive any treatment in their quitting attempts was not that attempting to quit without help is the most effective method, but simply because many of them had no access to help [5]. If they had access to treatment support, they might not have failed their quitting attempts. Consistently, those who succeeded in quitting may have achieved that much earlier if appropriate treatment support was accessible. Cessation services that target both quitting attempts and successful cessation are critical at a population level. Article 14 of the FCTC obligates the signatories to ensure a national cessation strategy and treatment guidelines [30] and recommends how to address this need in a way that is appropriate to the situation of the respective country using the existing resources. Most tobacco control measures in Bangladesh aim to prevent tobacco initiation and/or exposure to tobacco smoke in the environment. However, its achievement in reducing the overall prevalence of tobacco users through those control measures may be decelerated due to scarce cessation services and limited counselling facilities [31,32]. The treatment services that are available in Bangladesh focus mainly on smoking and rarely on SLT [33]. Cessation medications need to be made available and affordable [34]. Bulk buying and or inclusion of at least some cessation medicines on the national essential medicine list can assist [5].

Many of the factors related to past quitting attempts and future quitting intentions we identified in this study are similar to those identified in previous studies, with some differences [25,35,36]. The observed differences can be attributed to a range of aspects, including variations in survey design, sampling, participants’ characteristics, and contexts. However, overall, the literature suggests that comprehensive tobacco control measures, including tobacco taxation, proper implementation of smoke-free laws, anti-tobacco mass media campaigns, and barrier-free access to evidence-based cessation treatments, together, can work to prompt smokers to make quitting attempts and enhance their chances of quitting successfully [37]. While it is critical to increase the success rate among those who attempt to quit, getting more tobacco users to try to quit frequently is equally important.

The study has some limitations. Firstly, the GATS collects self-reported data; thus, the previous attempts and intentions to quit tobacco in the future may have been influenced by the recall and social desirability biases. Secondly, attempts and intentions to quit may be associated with the level of tobacco dependence [38,39]; however, our “user type” variable, which has two categories—occasional users and daily users—may not have captured the exact degree of dependence. It also would have been useful to examine the relationship between quitting duration and intention to quit in the future. However, since only a small proportion of participants mentioned the quitting duration in the form of months, weeks, and days, instead of a precise measure, we could not include it. Furthermore, data were collected from Bangladesh, and therefore the findings may not be generalisable to all settings. However, given that there are similarities among the neighbouring countries in terms of the pattern of tobacco use and related factors, the study findings may be partially generalizable to the sub-continent.

## 5. Conclusions

Smokers in Bangladesh were significantly more likely than SLT users to have made quitting attempts in the past 12 months and intend to quit in the future. Those who were both smoking and using smokeless tobacco were even more likely to report previous attempts and future intentions to quit. Those who had made quitting attempts in the 12 months before the survey were more likely than others to intend to quit in the future. The vast majority of the smokers and smokeless tobacco users who attempted to quit did not use any cessation treatment methods. Tailored interventions may increase quitting attempts. Tobacco cessation services need to be expanded and made affordable and available in all healthcare settings and communities.

## Figures and Tables

**Table 1:** Demographic features of the smokers and smokeless tobacco users in 2009 and 2017.

Variable	Smoking		Smokeless Tobacco Use	
	2009	2017	2009	2017
**Current users, %**	23.0	18.0	27.2	20.6
**Age in years, %**				
15–24	3.6	2.0	2.0	1.1
25–44	11.2	9.3	11.6	8.2
45–64	6.7	5.1	9.9	7.9
65+	1.5	1.6	3.7	3.5
**Age first started using tobacco, mean**	18.5	18.3	26.6	28.4
**Sex, %**				
Male	22.2	17.6	13.1	7.9
Female	0.8	0.4	14.1	12.7
**Education, %**				
No formal education	11.1	6.9	15.1	11.0
Less than Primary	4.2	3.7	4.5	3.9
Primary	2.1	2.3	3.1	2.0
Less than Secondary	3.6	2.9	2.9	2.4
Secondary or more	2.1	2.3	1.5	1.3
**Believe tobacco use is harmful for health, %**				
Yes	22.5	17.7	26.8	19.9
No	0.5	0.3	0.4	0.7
**Wealth quintiles, %**				
Lowest	5.6	2.4	6.8	1.8
Low	6.1	3.4	7.0	3.8
Middle	4.7	4.0	5.4	4.5
High	4.6	4.1	5.4	5.0
Highest	2.0	4.1	2.6	5.5
**Profession, %**				
Job (government/private)	1.8	2.1	1.1	0.9
Business	5.4	4.8	3.6	2.2
Farming/Agricultural work	8.9	5.0	5.4	2.6
Labourer	3.9	4.3	2.4	1.9
Homemaker/Unemployed/other	3.0	1.8	14.7	13.1
**Residence, %**				
Urban	5.6	4.4	5.9	3.7
Rural	17.4	13.6	21.3	16.9
**Quitting attempts among the current users during the 12 months prior to the survey, %**	45.3	43.4	26.9	29.9
**Current users intending to quit in the future, %**	68.0	66.0	48.7	51.2

**Table 2:** Multivariable Poisson regression (with robust variance) examining the associations between smoke and/or smokeless tobacco use and quitting attempts in the 12 months before the survey and intention to quit in the future.

Variable	Quitting Attempts in Past 12 Months aPR (95% CI) *n* = 12,632	Intention to Quit in Future aPR (95% CI) *n* = 12,577
**Tobacco type**		
Smokeless (ref)	1	1
Smoke	**1.38 (1.24–1.53)**	**1.09 (1.02–1.16)**
Both smoke and smokeless	**1.58 (1.41–1.77) §**	**1.19 (1.11–1.28) §**
**User type**		
Occasional users (ref)	1	1
Daily users	**0.79 (0.67–0.93)**	**0.76 (0.71–0.82)**
**Received counselling, NRT, Quitline, or traditional treatment in the past 12 months**		
No (ref)	1	1
Yes	**2.99 (2.86–3.12)**	**1.41 (1.34–1.50)**
**Age**		
15–24 (ref)	1	1
25–44	0.96 (0.84–1.09)	0.98 (0.90–1.06)
45–64	0.96 (0.83–1.10)	0.94 (0.86–1.03)
65+	0.90 (0.76–1.06)	**0.82 (0.74–0.92)**
**Sex**		
Male (ref)	1	1
Female	0.95 (0.83–1.09)	**0.62 (0.57–0.67)**
**Education**		
No formal education (ref)	1	1
Less than Primary	1.02 (0.94–1.11)	1.04 (0.99–1.10)
Primary	0.96 (0.86–1.07)	1.03 (0.96–1.10)
Less than Secondary	1.07 (0.97–1.17)	1.07 (1.01–1.13)
Secondary or more	1.10 (0.97–1.25)	1.07 (0.98–1.16)
**Wealth quintiles**		
Lowest (ref)	1	1
Low	1.06 (0.96–1.18)	1.02 (0.95–1.09)
Middle	1.04 (0.93–1.15)	1.04 (0.97–1.12)
High	1.05 (0.95–1.17)	1.04 (0.97–1.11)
Highest	0.97 (0.87–1.08)	1.07 (0.99–1.14)
**Profession**		
Job (government/private) (ref)	1	1
Business	1.04 (0.91–1.20)	1.09 (0.99–1.20)
Farming/Agricultural work	0.96 (0.83–1.10)	1.04 (0.95–1.15)
Labourer	0.89 (0.77–1.04)	1.02 (0.92–1.13)
Homemaker/Unemployed/other	1.03 (0.88–1.20)	1.15 (1.03–1.28)
**Residence**		
Urban (ref)	1	1
Rural	**0.94 (0.87–1.00)**	1.05 (0.99–1.10)
**Age first started using tobacco daily**	1.00 (0.99–1.01)	**1.01 (1.00–1.01)**
**Believe tobacco use is harmful to health**		
No (ref)	1	1
Yes	1.21 (0.94–1.56)	**1.21 (1.01–1.42)**
**Year**		
2009 (ref)	1	1
2017	1.04 (0.97–1.11)	1.00 (0.95–1.04)

aPR—adjusted prevalence ratio; §—attempted/intend to quit smoke/smokeless or both forms of tobacco.

**Table 3:** Multivariable Poisson regression (with robust variance) examining the associations between intention to quit in the future and quitting attempts during the 12 months before the survey.

Variable	Intention to Quit Smoking aPR (95% CI) *n* = 6415	Intention to Quit Smokeless Tobacco aPR (95% CI) *n* = 7320
**Attempted to quit in 12 months before the survey**		
No	1	1
Yes	**1.45 (1.38–1.53)**	**1.87 (1.76–1.98)**
**Tobacco type**		
Smokeless (ref)	-	1
Smoke (ref)	1	-
Both smoke and smokeless	0.96 (0.91–1.02)	0.94 (0.87–1.02)
**User type**		
Occasional users (ref)	1	1
Daily users	**0.81 (0.75–0.88)**	**0.80 (0.68–0.95)**
**Received counselling, NRT, Quitline or traditional treatment in the past 12 months**		
No (ref)	1	1
Yes	**1.14 (1.08–1.20)**	1.01 (0.91–1.11)
**Age**		
15–24 (ref)	1	1
25–44	0.97 (0.87–1.07)	0.93 (0.81–1.06)
45–64	0.99 (0.89–1.10)	**0.84 (0.73–0.97)**
65+	1.06 (0.93–1.21)	**0.67 (0.57–0.79)**
**Sex**		
Male (ref)	1	1
Female	**0.54 (0.40–0.73)**	**0.61 (0.54–0.68)**
**Education**		
No formal education (ref)	1	1
Less than Primary	1.04 (0.98–1.11)	1.01 (0.93–1.09)
Primary	1.03 (0.94–1.12)	1.05 (0.96–1.16)
Less than Secondary	1.01 (0.93–1.08)	**1.15 (1.06–1.25)**
Secondary or more	1.06 (0.95–1.18)	1.00 (0.89–1.14)
**Wealth quintiles**		
Lowest (ref)	1	1
Low	1.02 (0.93–1.11)	1.01 (0.91–1.12)
Middle	0.98 (0.90–1.06)	**1.14 (1.02–1.26)**
High	0.99 (0.92–1.08)	1.09 (0.98–1.20)
Highest	1.04 (0.95–1.13)	**1.14 (1.03–1.26)**
**Profession**		
Job (government/private) (ref)	1	1
Business	1.09 (0.96–1.23)	0.97 (0.84–1.12)
Farming/Agricultural work	1.05 (0.92–1.19)	0.91 (0.79–1.06)
Labourer	1.08 (0.95–1.23)	0.88 (0.75–1.04)
Homemaker/Unemployed/other	1.10 (0.96–1.26)	1.04 (0.89–1.21)
**Residence**		
Urban (ref)	1	1
Rural	**1.07 (1.01–1.14)**	1.06 (0.99–1.13)
**Age first started using tobacco daily ∆**	1.00 (0.99–1.01)	**1.01 (1.01–1.02)**
**Believe tobacco use is harmful for health**		
No (ref)	1	1
Yes	1.13 (0.92–1.39)	1.18 (0.94–1.47)
**Year**		
2009 (ref)	1	1
2017	0.97 (0.92–1.02)	1.01 (0.94–1.08)

aPR—adjusted prevalence ratio; ∆—the reference group is the “started smoking tobacco daily” for the intention to quit smoking model, and “started smokeless tobacco daily” for the intention to quit smokeless tobacco model.

## Data Availability

GATS data are publicly available from the WHO NCD microdata repository.
